# *trans*-Dichloridobis(triisopropyl­phosphine-κ*P*)palladium(II)

**DOI:** 10.1107/S1600536808018904

**Published:** 2008-06-28

**Authors:** Aleksandra Wisniewska, Katarzyna Baranowska, Jerzy Pikies

**Affiliations:** a Department of Inorganic Chemistry, Gdańsk University of Technology, 11/12 G. Narutowicz Street, 80952-PL Gdańsk, Poland

## Abstract

The title compound, [PdCl_2_(C_9_H_21_P)_2_], is a centrosymmetric mononuclear palladium(II) complex. The Pd^II^ atom, which lies on an inversion center, is in a square-planar geometry.

## Related literature

For *trans-*dichlorido-bis­(triphenyl­phosphine)palladium(II), see: Ferguson *et al.* (1982 ▶). For *trans-*dichlorido-bis­[diphenyl (cyclo­hexyl)phosphine]palladium(II), see: Meij *et al.* (2003 ▶). For *trans-*dichlorido-bis­[diphen­yl(*p*-tol­yl)phosphine]palla­dium(II), see: Steyl *et al.* (2006 ▶). For related literature, see: Baum *et al.* (2006 ▶); Bedford *et al.* (2003 ▶); Schultz *et al.* (1992 ▶).
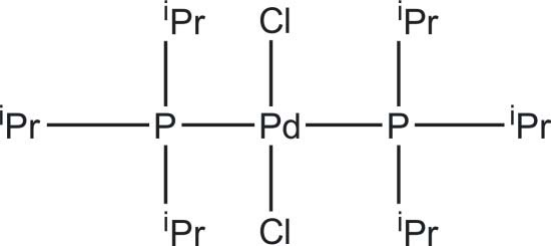


## Experimental

### 

#### Crystal data

[PdCl_2_(C_9_H_21_P)_2_]
*M* *_r_* = 497.76
Monoclinic, 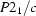

*a* = 8.0919 (3) Å
*b* = 8.9176 (4) Å
*c* = 16.1920 (6) Å
β = 92.552 (3)°
*V* = 1167.26 (8) Å^3^
*Z* = 2
Mo *K*α radiation
μ = 1.16 mm^−1^
*T* = 120 (2) K
0.15 × 0.09 × 0.02 mm

#### Data collection

Oxford Diffraction KM-4-CCD diffractometer
Absorption correction: analytical (*CrysAlis RED*; Oxford Diffraction, 2006 ▶) *T*_min_ = 0.791, *T*_max_ = 0.955
7043 measured reflections
2175 independent reflections
1985 reflections with *I* > 2σ(*I*)
*R*_int_ = 0.042

#### Refinement

*R*[*F* ^2^ > 2σ(*F* ^2^)] = 0.030
*wR*(*F* ^2^) = 0.089
*S* = 1.13
2175 reflections
112 parameters
H-atom parameters constrained
Δρ_max_ = 1.44 e Å^−3^
Δρ_min_ = −0.65 e Å^−3^

### 

Data collection: *CrysAlis CCD* (Oxford Diffraction, 2006 ▶); cell refinement: *CrysAlis RED* (Oxford Diffraction, 2006 ▶); data reduction: *CrysAlis RED*; program(s) used to solve structure: *SHELXS97* (Sheldrick, 2008 ▶); program(s) used to refine structure: *SHELXL97* (Sheldrick, 2008 ▶); molecular graphics: *ORTEP-3 for Windows* (Farrugia, 1997 ▶); software used to prepare material for publication: *WinGX* (Farrugia, 1999 ▶).

## Supplementary Material

Crystal structure: contains datablocks global, I. DOI: 10.1107/S1600536808018904/ci2618sup1.cif

Structure factors: contains datablocks I. DOI: 10.1107/S1600536808018904/ci2618Isup2.hkl

Additional supplementary materials:  crystallographic information; 3D view; checkCIF report

## Figures and Tables

**Fig. 1.**
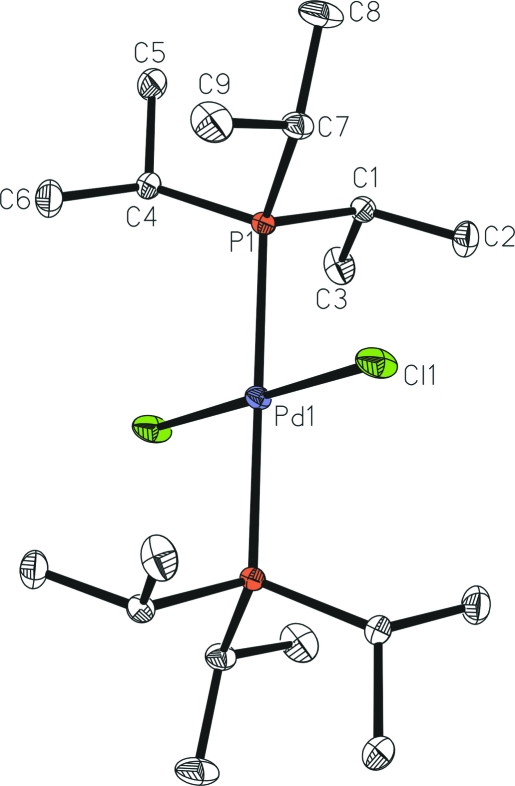
A view of the title molecule, showing the atom-labelling scheme. Displacement ellipsoids are drawn at the 50% probability level and H atoms have been omitted for clarity. Unlabelled atoms are related to labelled atoms by the symmetry operation (1-x, 1-y, -z).

**Fig. 2.**
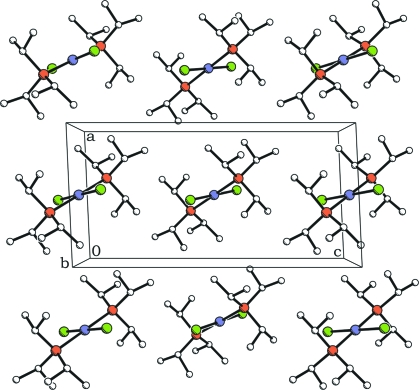
Crystal packing of the title compound, viewed approximately along the b axis.

## supplementary crystallographic information

### Comment

Expanding our work upon the reactivity of [(*R*_3_P)_2_MCl_2_] (*M* = Ni, Pd, Pt) towards *^t^*Bu_2_P–PLi–P*^t^*Bu_2_ (Baum *et al.*, 2006), we have studied the reaction of *^t^*Bu_2_P-PLi-P(*^t^*Bu)(SiMe_3_).2THF with [*trans*-(^i^Pr_3_P)_2_PdCl_2_] in a 1:1 molar ratio in THF. Unreacted [*trans*-(^i^Pr_3_P)_2_PdCl_2_] was isolated from the toluene solution of reaction product as yellow crystals.

The molecular structure of the title compound is shown in Fig.1. The mononuclear complex is centrosymmetric, with the Pd^II^ atom lying on an inversion centre. The geometry around the Pd^II^ atom is strictly square-planar. The Pd—P [2.3603 (6) Å] and Pd—Cl [2.3030 (6) Å] distances and P—Pd—Cl [89.92 (2)° and 90.18 (2)°] angles are typical for [*trans*-(*R*_3_P)_2_PdCl_2_] (Ferguson *et al.*, 1982; Meij *et al.*, 2003; Steyl *et al.*, 2006; Bedford *et al.*, 2003). The distances in [*cis*-(*R*_3_P)_2_PdCl_2_] differ significantly from those reported for [*trans*-(*R*_3_P)_2_PdCl_2_]. For [*cis*-(Me_3_P)_2_PdCl_2_], the related distances are 2.374 (3) Å (Pd—Cl, mean value) and 2.258 (2) Å (Pd—P, mean value) (Schultz *et al.*, 1992). The elongation of Pd—Cl distances in *cis* isomers compared to *trans *isomers is due to a strong *trans* effect of P*R*_3_ ligand in a position *trans* to Cl ligand. The shortening of Pd—P distances in *cis* isomers are caused by a lack of a second phosphine ligand in the *trans* position. The Cl ligand exerts only weak *trans* effect.

### Experimental

A solution of *^t^*Bu_2_P-PLi-P(*^t^*Bu)(SiMe_3_).2THF (139 mg, 0.285 mmol) in tetrahydrofuran (THF, 2 mL) was added dropwise to a suspension of yellow powder of [(^i^Pr_3_P)_2_PdCl_2_] (139 mg, 0.28 mmol) in THF (2 ml) at room temperature. The mixture turned red. After allowed to stand at room temperature for 1 d, the mixture was dried under vacuum at 1 mTorr for 1 h, and the residue was dissolved in toluene (4 ml) and filtered. The solution was kept at 277 K for 2d to obtain small yellow crystals of [*trans*-(^i^Pr_3_P)_2_PdCl_2_].

### Refinement

All H atoms were positioned geometrically and refined using a riding model, with C–H = 0.98 Å and U_iso_(H) = 1.2U_eq_(C) or 1.5U_eq_(methyl C). The highest residual electron-density peak is located 0.86 Å from atom Cl1.

### Crystal data

**Table:**

[PdCl_2_(C_9_H_21_P)_2_]	*F*_000_ = 520
*M**_r_* = 497.76	*D*_x_ = 1.416 Mg m^−^^3^
Monoclinic, *P*2_1_/*c*	Mo *K*α radiation λ = 0.71073 Å
Hall symbol: -P 2ybc	Cell parameters from 7947 reflections
*a* = 8.0919 (3) Å	θ = 2.3–32.5º
*b* = 8.9176 (4) Å	µ = 1.16 mm^−^^1^
*c* = 16.1920 (6) Å	*T* = 120 (2) K
β = 92.552 (3)º	Prism, colourless
*V* = 1167.26 (8) Å^3^	0.15 × 0.09 × 0.02 mm
*Z* = 2	

### Data collection

**Table:**

Oxford Diffraction KM-4-CCD diffractometer	2175 independent reflections
Monochromator: graphite	1985 reflections with *I* > 2σ(*I*)
Detector resolution: 8.1883 pixels mm^-1^	*R*_int_ = 0.042
*T* = 120(2) K	θ_max_ = 25.5º
0.75° wide ω scans	θ_min_ = 2.5º
Absorption correction: analytical(CrysAlis RED; Oxford Diffraction, 2006)	*h* = −9→9
*T*_min_ = 0.791, *T*_max_ = 0.955	*k* = −10→6
7043 measured reflections	*l* = −19→19

### Refinement

**Table:**

Refinement on *F*^2^	Secondary atom site location: difference Fourier map
Least-squares matrix: full	Hydrogen site location: inferred from neighbouring sites
*R*[*F*^2^ > 2σ(*F*^2^)] = 0.030	H-atom parameters constrained
*wR*(*F*^2^) = 0.089	*w* = 1/[σ^2^(*F*_o_^2^) + (0.0568*P*)^2^ + 0.2267*P*] where *P* = (*F*_o_^2^ + 2*F*_c_^2^)/3
*S* = 1.13	(Δ/σ)_max_ = 0.001
2175 reflections	Δρ_max_ = 1.44 e Å^−^^3^
112 parameters	Δρ_min_ = −0.65 e Å^−^^3^
Primary atom site location: structure-invariant direct methods	Extinction correction: none

### Special details

**Table:**

Geometry. All e.s.d.'s (except the e.s.d. in the dihedral angle between two l.s. planes) are estimated using the full covariance matrix. The cell e.s.d.'s are taken into account individually in the estimation of e.s.d.'s in distances, angles and torsion angles; correlations between e.s.d.'s in cell parameters are only used when they are defined by crystal symmetry. An approximate (isotropic) treatment of cell e.s.d.'s is used for estimating e.s.d.'s involving l.s. planes.
Refinement. Refinement of *F*^2^ against ALL reflections. The weighted *R*-factor *wR* and goodness of fit *S* are based on *F*^2^, conventional *R*-factors *R* are based on *F*, with *F* set to zero for negative *F*^2^. The threshold expression of *F*^2^ > σ(*F*^2^) is used only for calculating *R*-factors(gt) *etc*. and is not relevant to the choice of reflections for refinement. *R*-factors based on *F*^2^ are statistically about twice as large as those based on *F*, and *R*- factors based on ALL data will be even larger.

### Fractional atomic coordinates and isotropic or equivalent isotropic displacement parameters (Å^2^)

**Table:**

	*x*	*y*	*z*	*U*_iso_*/*U*_eq_	
Pd1	0.5	0.5	0	0.01758 (14)	
P1	0.62758 (8)	0.33350 (7)	0.09656 (4)	0.01777 (17)	
Cl1	0.53892 (10)	0.69501 (7)	0.09206 (4)	0.0358 (2)	
C1	0.4699 (3)	0.2487 (3)	0.16092 (14)	0.0218 (5)	
H1	0.529	0.1858	0.204	0.026*	
C2	0.3743 (3)	0.3692 (3)	0.20534 (17)	0.0322 (6)	
H2A	0.3178	0.4347	0.1645	0.048*	
H2B	0.4512	0.4288	0.2404	0.048*	
H2C	0.2925	0.3217	0.2397	0.048*	
C3	0.3525 (4)	0.1470 (3)	0.11119 (18)	0.0352 (7)	
H3A	0.2641	0.1132	0.1462	0.053*	
H3B	0.4134	0.0599	0.0916	0.053*	
H3C	0.3043	0.2023	0.0637	0.053*	
C4	0.7288 (3)	0.1727 (3)	0.04740 (15)	0.0222 (5)	
H4	0.639	0.1221	0.0134	0.027*	
C5	0.7990 (4)	0.0510 (3)	0.10597 (17)	0.0336 (6)	
H5A	0.8222	−0.0396	0.0742	0.05*	
H5B	0.7182	0.0277	0.1474	0.05*	
H5C	0.9015	0.0871	0.1337	0.05*	
C6	0.8569 (4)	0.2198 (3)	−0.01420 (17)	0.0355 (7)	
H6A	0.9591	0.2505	0.0159	0.053*	
H6B	0.8136	0.304	−0.0475	0.053*	
H6C	0.8802	0.1352	−0.0505	0.053*	
C7	0.7729 (3)	0.4281 (3)	0.17166 (15)	0.0248 (5)	
H7	0.7088	0.512	0.1959	0.03*	
C8	0.8372 (4)	0.3339 (3)	0.24483 (18)	0.0393 (7)	
H8A	0.9122	0.2566	0.2254	0.059*	
H8B	0.7439	0.2861	0.2711	0.059*	
H8C	0.8968	0.3985	0.285	0.059*	
C9	0.9156 (5)	0.5020 (3)	0.1284 (2)	0.0403 (9)	
H9A	0.9727	0.5725	0.1663	0.06*	
H9B	0.8723	0.5562	0.0794	0.06*	
H9C	0.9936	0.4249	0.1116	0.06*	

### Atomic displacement parameters (Å^2^)

**Table:**

	*U*^11^	*U*^22^	*U*^33^	*U*^12^	*U*^13^	*U*^23^
Pd1	0.0209 (2)	0.0166 (2)	0.01496 (18)	−0.00014 (9)	−0.00173 (12)	−0.00049 (8)
P1	0.0189 (3)	0.0187 (3)	0.0156 (3)	0.0011 (2)	−0.0004 (2)	0.0009 (2)
Cl1	0.0575 (5)	0.0223 (3)	0.0259 (3)	0.0064 (3)	−0.0155 (3)	−0.0074 (3)
C1	0.0226 (12)	0.0224 (11)	0.0205 (12)	0.0007 (10)	0.0030 (10)	0.0034 (10)
C2	0.0311 (14)	0.0334 (14)	0.0329 (14)	0.0026 (13)	0.0120 (12)	−0.0013 (12)
C3	0.0326 (15)	0.0392 (16)	0.0344 (15)	−0.0152 (13)	0.0068 (12)	−0.0051 (12)
C4	0.0226 (12)	0.0216 (12)	0.0225 (12)	0.0035 (10)	0.0023 (10)	−0.0014 (10)
C5	0.0391 (17)	0.0292 (14)	0.0329 (15)	0.0120 (14)	0.0053 (13)	0.0000 (13)
C6	0.0354 (16)	0.0381 (16)	0.0341 (15)	0.0051 (13)	0.0129 (13)	0.0002 (12)
C7	0.0252 (13)	0.0262 (13)	0.0225 (12)	−0.0011 (11)	−0.0049 (10)	−0.0019 (10)
C8	0.0494 (18)	0.0365 (15)	0.0301 (15)	0.0017 (14)	−0.0196 (13)	−0.0004 (12)
C9	0.0307 (19)	0.052 (2)	0.0378 (19)	−0.0199 (12)	−0.0029 (16)	−0.0036 (12)

### Geometric parameters (Å, °)

**Table:**

Pd1—Cl1^i^	2.3030 (6)	C4—C5	1.533 (4)
Pd1—Cl1	2.3030 (6)	C4—H4	1
Pd1—P1	2.3603 (6)	C5—H5A	0.98
Pd1—P1^i^	2.3603 (6)	C5—H5B	0.98
P1—C1	1.845 (2)	C5—H5C	0.98
P1—C4	1.849 (2)	C6—H6A	0.98
P1—C7	1.856 (2)	C6—H6B	0.98
C1—C3	1.518 (4)	C6—H6C	0.98
C1—C2	1.523 (3)	C7—C8	1.525 (4)
C1—H1	1	C7—C9	1.528 (4)
C2—H2A	0.98	C7—H7	1
C2—H2B	0.98	C8—H8A	0.98
C2—H2C	0.98	C8—H8B	0.98
C3—H3A	0.98	C8—H8C	0.98
C3—H3B	0.98	C9—H9A	0.98
C3—H3C	0.98	C9—H9B	0.98
C4—C6	1.529 (3)	C9—H9C	0.98
			
Cl1^i^—Pd1—Cl1	180.00 (3)	C6—C4—H4	105.2
Cl1^i^—Pd1—P1	89.82 (2)	C5—C4—H4	105.2
Cl1—Pd1—P1	90.18 (2)	P1—C4—H4	105.2
Cl1^i^—Pd1—P1^i^	90.18 (2)	C4—C5—H5A	109.5
Cl1—Pd1—P1^i^	89.82 (2)	C4—C5—H5B	109.5
P1—Pd1—P1^i^	180	H5A—C5—H5B	109.5
C1—P1—C4	104.84 (11)	C4—C5—H5C	109.5
C1—P1—C7	104.47 (11)	H5A—C5—H5C	109.5
C4—P1—C7	110.77 (12)	H5B—C5—H5C	109.5
C1—P1—Pd1	109.81 (8)	C4—C6—H6A	109.5
C4—P1—Pd1	113.07 (8)	C4—C6—H6B	109.5
C7—P1—Pd1	113.18 (8)	H6A—C6—H6B	109.5
C3—C1—C2	110.7 (2)	C4—C6—H6C	109.5
C3—C1—P1	112.16 (17)	H6A—C6—H6C	109.5
C2—C1—P1	110.86 (17)	H6B—C6—H6C	109.5
C3—C1—H1	107.6	C8—C7—C9	110.8 (2)
C2—C1—H1	107.6	C8—C7—P1	116.30 (18)
P1—C1—H1	107.6	C9—C7—P1	111.43 (19)
C1—C2—H2A	109.5	C8—C7—H7	105.8
C1—C2—H2B	109.5	C9—C7—H7	105.8
H2A—C2—H2B	109.5	P1—C7—H7	105.8
C1—C2—H2C	109.5	C7—C8—H8A	109.5
H2A—C2—H2C	109.5	C7—C8—H8B	109.5
H2B—C2—H2C	109.5	H8A—C8—H8B	109.5
C1—C3—H3A	109.5	C7—C8—H8C	109.5
C1—C3—H3B	109.5	H8A—C8—H8C	109.5
H3A—C3—H3B	109.5	H8B—C8—H8C	109.5
C1—C3—H3C	109.5	C7—C9—H9A	109.5
H3A—C3—H3C	109.5	C7—C9—H9B	109.5
H3B—C3—H3C	109.5	H9A—C9—H9B	109.5
C6—C4—C5	110.8 (2)	C7—C9—H9C	109.5
C6—C4—P1	113.17 (18)	H9A—C9—H9C	109.5
C5—C4—P1	116.20 (17)	H9B—C9—H9C	109.5

Symmetry codes: (i) −*x*+1, −*y*+1, −*z*.
